# Fertility awareness and intentions among young adults in Greece

**DOI:** 10.48101/ujms.v126.8148

**Published:** 2021-12-31

**Authors:** Ioanna Lardou, Ioannis Chatzipapas, Michail Chouzouris, Panos Xenos, Nikolaos Petrogiannis, Dimitrios Tryfos, Stephanos Chandakas, Themos Grigoriadis, Lina Michala

**Affiliations:** a 1st Department of Obstetrics and Gynaecology, National and Kapodistrian University of Athens, Alexandra General Hospital, Athens, Greece; b Department of Statistics and Insurance Science, University of Piraeus, Athens, Greece; c Naval and Veterans Hospital of Athens, Athens, Greece; d Mitera General Hospital, Athens, Greece

**Keywords:** Fertility awareness, assisted reproductive techniques, planned parenthood, age-related fertility, educational programs

## Abstract

**Background:**

Greece has a mean age of first motherhood at 31.5 years, higher than the European average age of 29.4. Delaying conception, however, may be an important non-reversible cause of infertility. The aim of this study was to identify possible knowledge deficits regarding fertility in young adults.

**Methods:**

This was an online survey of young adults, regarding information on intention to parenthood and knowledge on issues affecting fertility. This study was conducted from February to December 2020, aiming for a representative sample of Greek men and women aged 18 and 26 years. The questionnaire was designed by a multidisciplinary group based on the Cardiff Fertility Knowledge Scale, which contained 22 multiple-choice or Likert-scale questions.

**Results:**

We obtained responses from 1875 young adults, whose mean age was 22.1 years. About 91.8% of men and 94.0% of women declared an intention to have children, out of which 44.0% wanted to have two and 29.0% three children. About 52.0 and 50.8% men and women, respectively, aimed to start a family between 31 and 35 years. Residents of rural areas and those with a lower education level more likely aimed to have children before the age of 30. The most prevalent answers for age of ideal parenthood were between 26 and 30 years for a woman and 31–35 years for a man. Smoking, alcohol consumption and sexually transmitted infections were identified as factors affecting both female and male fertility. Half of men and women, respectively, overestimated general success rates of reproductive techniques.

**Conclusion:**

The knowledge of fertility, particularly with regards to assisted reproductive techniques’ success rates, may be overestimated as more young adults plan for having children after the age of 30.

## Introduction

According to recent data, Greece is among the countries presenting the highest rates of first childbirth among women at age 40 and over, estimated at 5.3%, with the mean age of motherhood steadily increasing and is currently 31.5 years, while the European average age is 29.4 years (1).

The main reasons for delayed childbearing include a competitive work environment, unemployment, immigration, reduced income, increased cost of raising children and limited access to healthcare services (2). Furthermore, women may prioritize financial independence and career development over the desire for childbirth (3). Greece has an unfavourable work environment for employed parents, at least when compared with other European countries. According to the European Institute of Gender Equality, 71% of Greek women are eligible for parental leave, as opposed to the European average of 90%. Restrictions particularly apply to those who are self-employed. Greek men are further underprivileged, as only 64% can obtain parental leave, compared with 88% of their European counterparts (4).

However, deferring parenthood may affect the ability to become a parent or to achieve the desired family size. Although social fertility preservation and assisted reproductive techniques (ARTs) may offer a solution, they provide by no means a guarantee. Still, the media are overwhelmed by news of celebrities and social influencers who have their children during the fifth or- sometimes sixth- decade of life (5). This information is rarely balanced by truthful recounts of the obstacles that some of these women may have faced to achieve a perceived happy outcome.

Promoting reliable information and realistic expectations regarding the reproductive ability should empower young people to make true informed choices about their future fertility, thus deciding or not to delay childbearing. A recent report on fertility awareness among medical students in three European countries showed that Greek students were relatively realistic about the prospects of fertility decline with age. This, however, may reflect the level of information that is obtained in the obstetrics and gynaecology curriculum, rather than knowledge and attitudes applicable to the general population (6). The aim of this study was to identify possible knowledge deficits in young adults in the general population in order to label areas of improvement in secondary education and possibly introduce public awareness strategies.

## Methods

This was an online survey of fertility awareness among young men and women aged 18–26 years in Greece.

### Development of the questionnaire

A multidisciplinary team, consisting of obstetricians and gynaecologists, fertility specialists, academic teachers and statisticians, discussed the aim of the study and the formulation of a questionnaire. A thorough literature search was performed to identify similar studies and questionnaires that could be used as a basis of the study. We based the questions on the Cardiff Fertility Knowledge Scale (7, 8), but opted for multiple choice and Likert scale questions rather than true or false options.

The final questionnaire was anonymous and comprised of 22 questions. Out of them, five questions were on demographics (age, municipality of residence, educational level and work status), four questions regarding the intention to have children, four questions regarding factors that affect male and female fertility, along with the ideal age to father or bear a child and finally nine questions regarding ART, including egg donation, egg freezing and success rates of ART, in general, and in women over the age of 40 years.

The questionnaire was piloted for brevity and clarity in a sample of 20 young adults, and final corrections were made accordingly. It was then transcribed in an electronic format via google forms platform for easier dissemination.

Cronbach’s alpha coefficient for the questionnaire omitting the five questions regarding demographics- was 0.79.

### Ethical approval

The study protocol received ethical approval from the scientific board of Alexandra General Hospital (Reference No. 693/02-09-2019). At the onset of the electronic form, we included information for the participant and an option to proceed to the questionnaire after checking an online consent form. The questionnaire was anonymous and did not ask for sensitive personal information.

### Sample size and Recruitment

In order to achieve a representative sample of the relevant population, we calculated the minimum number of participants, so as to achieve a 95% confidence interval and a margin of error of 3%. We thus aimed for a minimum sample of 1,067 (9).

An online survey was conducted for a period of 10 months from February to December 2020. Eligibility criteria were reading knowledge of Greek and age between 18 and 26 years. Data collection followed the ethical considerations and data privacy protocols while participants gave consent via an online privacy statement. In order to achieve representativeness of the Greek population, quotas were used for geographical regions. We advertised through posters that included a QR code linking to the online questionnaire. We particularly approached university students and military recruits, and generally encouraged participants to disseminate the questionnaire link to their social groups. A total of 2014 participated in the study, of which 139 were excluded because they were 27 years old or above.

### Statistical analysis

Descriptive statistics were used to describe demographic variables and fertility knowledge. Categorical data were compared using chi-squared tests. T-tests were used to compare scores of knowledge depending on the gender. A *P* value of <0.05 was considered to be statistically significant. We used the SPSS (version 25.0) for analysis.

## Results

Our sample consisted of 1,875 participants, of which 1,133 (60.4%) were women and 742 (39.6%) men. Out of them, 21 (1.1%) already had children, and their demographics are shown in Table 1. The place or residence was defined as urban when the population was over 10,000 according to the 2011 census (10).

**Table 1:** Demographics.

Age (mean)	Men (*n* = 742)		Women (*n* = 1,133)		Total (*n* = 1,875)	
	22.7		21.7		22.1	
	*n*	%	*n*	%	*n*	%
18	45	6.1	152	13.4	197	10.5
19	48	6.5	137	12.1	185	9.9
20	76	10.2	143	12.6	219	11.7
21	54	7.3	128	11.3	182	9.7
22	85	11.5	132	11.7	217	11.7
23	126	17	121	10.7	247	13.2
24	114	15.4	107	9.4	221	11.8
25	105	14.2	110	9.7	215	11.5
26	89	12	103	9.1	192	10.2
**Place of residence**						
Urban	649	87.5	1,033	91.2	1,682	89.7
Rural	82	11.1	88	7.8	170	9.1
Abroad	10	1.3	7	0.6	17	0.9
**Education**						
Middle school graduate	4	0.5	3	0.3	7	0.4
High school graduate	99	13.3	126	11.1	225	12
University student	596	80.3	970	85.6	1,566	83.5
**Working status**						
Student	362	48.8	766	67.6	1,128	60.2
Unemployed	65	8.8	31	2.7	96	5.1
Employed (full or part time)	291	39.2	285	25.2	576	30.7

### Intention of having children

About 91.8% of men and 94.0% of women revealed that they want to have children in the future, and out of them, 120 (6.4%) would want to have one, 821 (43.8%) two, 375 (20.0%) three and 126 (6.7%) more than three children. There was no statistical difference between men and women in their intention.

Subsequently, when asked about the likely age they would have children, 52.0 and 50.8% of men and women, respectively, aimed to have children between 31 and 35 years. Those who wanted to have more than three children planned to have them before the age of 30. However, there were 224 participants (11.9%), of which 118 women, who wished to have three or more children after the age of 30.

When comparing participants according to place of residence, those residing in a rural area were more likely to aim to have children before the age of 30 than those residing in urban centres (48.0 vs 40.0%, *P* = 0.014). Similarly, secondary school graduates were statistically significantly more likely to want to have children under the age of 30, in comparison with university students or graduates (53.2 vs 37.9%, *P* < 0.001). There was no statistically significant difference in the number of children planned, depending on the place of residence or education level.

### Knowledge on fertility and comparison to age of planned parenthood

**Figure 1:**
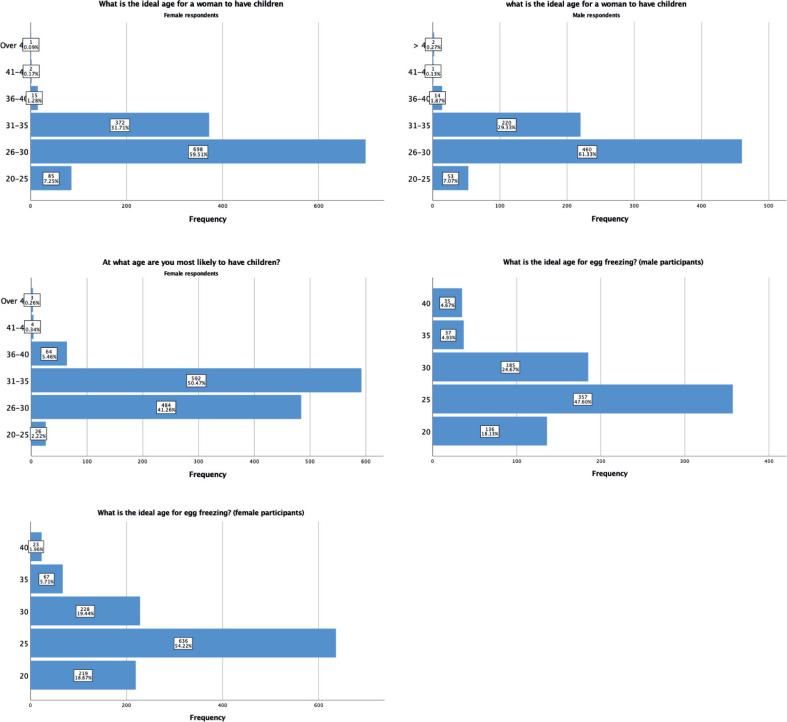
Fertility.

Next, participants were asked about the ideal age to have children, where the most prevalent answers were between 26 and 30 years for a woman (60.0%) and 31–35 years for a man (47.0%). Slightly more men than women agreed that the ideal age for a woman to have children is between 26 and 30 years of age (460- 61.3%, vs 698 -59.5% respectively). However, 41.2% of women (484) were seeing themselves likely to have children in this age margin, and 56.0% were more likely to have children after the age of 30 (**Figure 1**).

Following on, participants were asked to rate factors that affect the fertility of men and women, the results of which are listed in Tables 2 and 3.

**Table 2:** Men’s knowledge on factors affecting female fertility.

	Not at all (%)	2 (%)	3 (%)	4 (%)	5 (%)	6 (%)	Very significantly (%)	I don’t know (%)
Smoking	2.4	7.5	8.5	13.3	18.8	11.5	35.9	2.1
Obesity	2.7	8.3	10.0	16.5	20.3	12.9	23.9	5.5
Daily consumption of alcohol	1.3	5.3	5.1	10.7	16.7	14.9	44.1	1.9
Not using condoms in the past	13.9	8.0	10.0	10.7	15.3	12.5	19.3	10.3
Previous usage of the COC^a^	5.6	9.7	9.9	15.2	18.1	12.5	19.1	9.9
History of sexually transmitted infections	2.3	5.9	6.4	11.5	17.5	16.3	34.0	6.3
Previous abortion(s)	1.7	6.1	4.4	8.9	16.8	19.7	36.8	5.5
Age	2.4	6.4	4.0	8.7	17.9	21.9	36.9	1.9

a , COC: combined oral contraceptives.

**Table 3:** Women’s knowledge on factors affecting female fertility.

	Not at all (%)	2 (%)	3 (%)	4 (%)	5 (%)	6 (%)	Very Significantly (%)	I don’t know (%)
Smoking	1.0	13.2	9.8	12.3	14.0	8.2	35.0	6.5
Obesity	3.5	13.1	12.0	12.6	15.4	11.4	20.5	11.4
Daily usage of alcohol	0.7	10.0	6.8	9.3	11.5	12.4	44.9	4.3
Not using condoms in the past	25.9	10.2	6.7	9.4	9.0	5.6	11.1	22.1
Previous usage of the COC^a^	13.5	17.4	13.6	10.9	9.5	5.5	9.0	20.6
History of STIs^b^	2.7	11.6	9.5	13.1	12.9	11.7	24.9	13.6
Previous abortion(s)	3.0	12.2	9.6	11.7	12.4	14.9	28.1	8.0
Age	2.6	9.6	8.1	12.4	16.5	17.1	30.9	2.7

a , COC: combined oral contraceptives

b , STIs: sexually transmitted infections.

### Knowledge on ART

With regards to ART success rates, 24.0 and 30.0% of men and women, respectively, correctly approximated general ART cycle success rates at 30% (11). Nevertheless, 48.0 and 43.0% of men and women, respectively, overestimated general success rates, with 214 answering that this would amount to 90%. When asked about ART success rates per cycle in women over 40 years of age, 41.0% of men and 37.0% of women overestimated them to be over 30%, whereas 41.0% and 44.0% of men and women, respectively, correctly identified them to be in the 10–20% range.

Finally, 32.9% of women would consider egg freezing and 25.3% of men would recommend this to their partner. For the remainder, the most important reason for avoiding it was mostly the cost for women and a preference for natural conception for men. The results of the Likert scale are shown in Tables 4 and 5.

**Table 4:** Reasons to avoid egg freezing (male participants).

	Not at all (%)	2 (%)	3 (%)	4 (%)	5 (%)	6 (%)	Extremely (%)	I don’t know (%)
Cost	4.8	9.7	9.6	12.8	16.3	15.9	22.3	8.7
Impact on health	11.6	10.5	11.5	13.9	15.3	13.2	15.7	8.3
Lack of time	18.4	19.7	14.4	12.8	10.4	7.6	8.3	8.4
Prefer to conceive naturally	4.1	8.5	7.9	13.2	16.5	17.5	26.4	5.9
Uncertain about result	3.9	10.5	10.5	15.5	19.6	17.1	16.1	6.8
Worried about what others would think/say	13.3	9.9	10.7	12.8	14.0	14.4	17.1	7.9

**Table 5:** Reasons to avoid egg freezing (women participants).

	Not at all (%)	2 (%)	3 (%)	4 (%)	5 (%)	6 (%)	Extremely (%)	I don’t know (%)
Cost	1.4	7.8	8.6	11.0	15.3	11.9	32.0	11.9
Impact on health	12.7	11.0	9.2	11.8	13.2	9.7	19.1	13.2
Lack of time	24.1	21.0	14.4	11.5	8.3	3.6	4.7	12.4
Prefer to conceive naturally	3.5	9.7	8.7	11.0	15.4	15.1	29.2	7.3
Unsure of result	3.2	10.1	13.1	15.1	17.0	15.7	18.3	7.5
Worried about what others would think or say	21.8	13.0	11.8	12.9	10.9	9.0	12.8	7.8

## Discussion

This study focuses primarily on exploring tendencies and perceptions of young people in Greece in relation to reproductive health knowledge and assisted reproduction. We decided on an internet-based survey, as it is a convenient and effective way of reaching large numbers of younger participants, who in their majority will be computer native.

A sample size of 1,875 was well above our initial recruitment intention. We can thus draw important, generalizable conclusions. Young men remained relatively underrepresented in the sample. This is often the case in surveys where female responders may be more willing to participate (12, 13). Still, the number of men included in the study was deemed adequate.

With regards to intention to have children, participants overwhelmingly stated they wanted to have offsprings in the future, with almost half of them hoping to have two. Almost 27% stated that they would consider having three or more children. About half were thinking that they were more likely to start a family between the ages of 31 and 35 years. This was similar for both men and women, with 11.4% of women planning to have at least three children despite starting a family after the age of 30. This may demonstrate an overoptimistic approach on their ability to carry a pregnancy when into their late 30s or 40s.

About 5% of women and 10% of men were seeing themselves having children after the age of 36 years and a very small minority after the age of 40. Again, this proportion of young people may not be fully aware of age-related fertility decline. Still, this proportion was much lower than what was seen in a similar survey in the UK and Denmark, where nearly one-fifth of the women and one-third of the men desired a first child at or after 35 years (14).

Despite their intentions, about 60.0% of respondents in this study correctly acknowledged the ideal age of childbearing for a woman to be under 30 years. Age was also identified as a significant factor affecting female fertility by both men and women, with only 4.0 and 5.0% of men and women, respectively, stating that they either did not know or felt that age had any effect on fertility.

Smoking and alcohol consumption along with obesity were all identified as potentially detrimental factors for female and male fertility. Similarly, history of sexually transmitted infections (STIs) or previous abortions were considered to play a significant role in male and female fertility. It is, however, notable that prior use of condoms was among the least stated factors affecting either male or female fertility. Usage of condoms prevents STIs, and as such, we expected it to be identified as an important factor interfering with future fertility. It may, however, be that respondents did not fully appreciate the reasoning of the question. In future questionnaires, we could consider incorporating an explanatory sentence on this or adding further questioning on alternative ways to prevent STI transmission, such as frequent testing for highly prevalent STIs or having long-term, stable sexual relationships.

According to similar studies from other parts of the world, fertility awareness tends to be low to moderate (15, 16) irrespective of gender (17). Men, in particular, appear to have significant gaps in their knowledge of factors affecting their fertility, although they were more aware of modifiable risk factors, such as STIs and smoking cigarettes, as opposed to fixed and health-related factors, such as delayed puberty, diabetes and cardiovascular disease (18).

With regards to egg freezing, about half of participants correctly stated that this should ideally take place prior to 30 years of age. However, only about one-third had a clear positive attitude towards this. With fertility declining with age and women favouring career over childbearing, social egg freezing is an appealing option for fertility preservation. More women are storing their oocytes to maintain the potential to have a baby in the future. However, they tend to proceed to egg freezing when they are already in their later 30s when the success rates of the method are limited (19). Furthermore, younger women are disadvantaged by the current legislated limit of 10 years’ duration of storage (20).

In this study, the main barrier to social egg freezing was cost. A proportion of participants also mentioned that they would prefer natural methods of conception or that they were uncertain about results. This proportion was similar to the number of undecided participants regarding the recourse to reproductive fertility methods, in general. Lack of time had the least impact on their decision to future egg freezing.

With regards to ART cycle success rates, about one-third of participants correctly appreciated this to be in the range of 30% (11). Furthermore, almost half accurately recognized reduced success rates per ART cycle in women over the age of 40 (21). The remainder, however, tended to be overoptimistic about the ability of ART to overcome infertility. Likewise, past studies of highly educated young adults in Europe and America found that they were not sufficiently aware of age-related female infertility and falsely believed that ART will overcome any fertility problems, even those associated with age (22–25). Similar results were found in a study of Chinese university students (13).

The majority of participants were living in urban centres, which is typical of the current demographic distribution in the country (24) and, as expected, only 1% of participants stated that they already had children (25). Nevertheless, there were limitations, in that eight out of 10 participants (83.5%) were students or university graduates, a proportion that is higher than what is typical for young Greeks (26). Also, less number of participants were unemployed (27), possibly due to the fact that they were defining themselves as students. It is possible, therefore, that participants favoured career over family, more so than in the general population of young adults, and this may explain a tendency to postpone parenthood after professional goals have been fulfilled. However, a higher level of education would also suggest more opportunities for exposure to information regarding reproductive health, and this indicates a likely failing in the education system. Fertility education should be part of the core curriculum in schools in order to ensure children and young people have a good foundation on reproductive health and choices.

Several countries have already introduced relevant educational programmes. For example, the British Fertility Society, in partnership with the Royal College of Obstetricians and Gynaecologists, other learned societies and non-profit institutions introduced the ‘Fertility Education Initiative’, aiming at providing accurate information about fertility at schools and promoting fertility awareness (28). On a similar note, the Center for Disease Control introduced the ‘Reproductive Life Plan (RLP)’ to promote preconceptional health for those intending to have children in the future (29). Finally, the Australian government has funded a public education programme named ‘Your Fertility’, aiming to improve knowledge among health professionals and the public (30). The project has also focused on introducing teaching resources for primary and secondary schools, with supporting online learning tools for teachers, including an e-learning module and class handout material (31).

Social media and internet websites can also influence and inform the public about infertility (5), as shown by the study that assesses an evidence-based website on RLP called ‘reproduktivlivsplan.se’ (32). However, many accounts currently available are created by patient groups or the private sector, and thus, may provide skewed, non-evidence-based information. Hopefully, more learned societies will create social media accounts in the future in order to provide accurate, yet approachable material. We would also expect relevant societies in Greece to provide healthcare providers with materials to help young people form reproductive goals, improve future fertility or advice on fertility preservation, following the successful example of the Swedish RLP model (33).

In conclusion, the results of this project provide a snapshot of what are the current knowledge and intentions of young men and women in Greece regarding childbearing and fertility. Although there are intentions for childbearing before the age of 30, and despite adequate understanding of the limitations of ART on improving fertility, there are still a significant proportion of young adults who lack the necessary information about factors leading to declining fertility. Although social and financial reasons may be at the heart of postponed motherhood, improving fertility awareness through specific educational programmes can provide with the necessary knowledge to make informed decisions about deferring or not childbearing.

With this in mind, we suggest the introduction of relevant educational programmes for the general public, including the incorporation of fertility awareness in the sexual and relations education curriculum, following the Australian and Swedish paradigm.
